# Circulating 25-hydroxyvitamin D levels and hypertension risk after adjusting for publication bias

**DOI:** 10.1186/s40885-022-00196-4

**Published:** 2022-05-15

**Authors:** Jong-Myon Bae

**Affiliations:** Department of Preventive Medicine, Jeju National University College of Medicine, 102 Jejudaehak-ro, Jeju-si, Jeju Province 63243 Korea

**Keywords:** Vitamin D, Hypertension, Publication bias, Systematic review, Meta-analysis

## Abstract

**Background:**

Previous systematic reviews reported that serum vitamin D deficiency was associated with risk of hypertension. The aim was to conduct a meta-epidemiological analysis for evaluating the potential effects of publication bias.

**Methods:**

The selection criterion was defined as a follow-up study for evaluating the association between circulating 25-hydroxyvitam D level and hypertension risk in adults. A funnel plot and Egger’s test were used to detect a publication bias. If a publication bias was identified, trim-and-fill analysis (TFA) with linear estimator was performed to estimate a summary relative risk (sRR).

**Results:**

The meta-analysis of 13 cohorts resulted in the lower the vitamin D, the higher the risk of hypertension statistically significant (sRR, 1.22; 95% confidence interval [CI], 1.05 to 1.41). But The *P*-value of Egger’s test (=0.015) and asymmetry of the funnel plot showed that there was a publication bias. TFA resulted in that statistical significance disappeared in the association between vitamin D level and hypertension risk in total cohorts (filled sRR, 1.03; 95% CI, 0.89 to 1.18) as well as men and women cohorts.

**Conclusions:**

The publication bias-adjusted results by TFA had no statistically significant association between vitamin D levels and the risk of hypertension. The significant results in previous systematic reviews might be interpreted as due to publication bias.

## Background

Vitamin D is associated with the renin-angiotensin system, vascular inflammation, and calcification [1–4]. Based on these facts, the hypothesis that circulating vitamin D level would be associated with a risk of hypertension has been consistently suggested [5].

Four systematic reviews evaluating the hypothesis were published [6–9]. They all concluded that there was a statistical significance between lower level of circulating 25 hydroxyvitamin D [25(OH)D] and risk of hypertension (Table 1). However, as shown in Table 1, the existing systematic reviews did not sufficiently consider the publication bias through funnel plot and Egger’s test.

**Table 1:** Summary of previous systematic reviews for evaluating the association between serum 25(OH) vitamin D levels and hypertension risk

Author (year)	Pittas et al. [6] (2010)	Burgaz et al. [7] (2011)	Kunutsor et al. [8] (2013)	Qi et al. [9] (2017)
Search to	Nov 2009	Nov 2010	Nov 2012	May 2015
Selected cohort studies	2	3	6	7
Analysis strategy	Lowest to Highest	Highest to Lowest	Top to bottom	Deficiency to Sufficient
sRR (95% CI)	1.76 (1.27–2.44)	0.73 (0.63–0.84)	0.68 (0.60–0.77)	1.24 (1.08–1.41)
I-squared	0.0	–	–	72.1
Funnel plot	NC	NC	CN	CN
Egger test (*P*-value)	NC	0.36	0.08	CN

*sRR* summary relative risk, *CI* confidence intervals, *NC* not considered, *CN* considered but did not report results

Meanwhile, vitamin D deficiency is more common in women [10]. Therefore, the risk of hypertension due to vitamin D deficiency can be expected to vary by sex. But the existing systematic reviews in Table 1 did not present the summary relative risk (sRR) of subgroup analysis by sex. The aim was to conduct a meta-epidemiological analysis [11] for evaluating the association between serum 25(OH)D levels and hypertension risk in men and women after adjusting publication bias.

## Methods

### Selection strategies

A total of 11 studies selected for meta-analysis by four systematic reviews in Table 1 were selected as potential study subjects [9, 12–21]. Considering that the most recently published year among these was 2017 [9], it was necessary to secure additional articles to be selected as of November 30, 2020. Accordingly, a list of articles that cited previously selected 11 papers was made using the ‘cited by’ option provided by PubMed [22].

Then, each article was evaluated whether it satisfies the selection criterion such as a follow-up study for evaluating the association between circulating 25(OH)D level and hypertension risk in adults. After selecting the articles that satisfy the selection criterion, it was checked whether the participants duplicate among the selected studies. If there were overlaps in the study participants, a study with a larger number of hypertensive patients was selected.

### Statistical analysis

The sRR adjusted for the most confounders in each study were extracted. The lowest to highest sRR values were unified to determine the risk of hypertension due to vitamin D deficiency. If the sRR was presented with the lowest circulating 25(OH)D level as the reference, the inverse was used such that the highest level as the reference.

Heterogeneity of studies was assessed with I-squared value (%). Random effects model meta-analysis was performed [23]. Publication bias was examined by funnel plot and Egger’s test [24, 25]. If a publication bias was identified, trim-and-fill analysis (TFA) with linear estimator was performed using fixed-random effects model in STATA ver. 14.2 (StataCorp, TX, USA) [26, 27]. The statistical significance level was set to 0.05. The author followed the guideline for reporting meta-epidemiological methodology research [11].

## Results

### Final selection

**Fig. 1:**
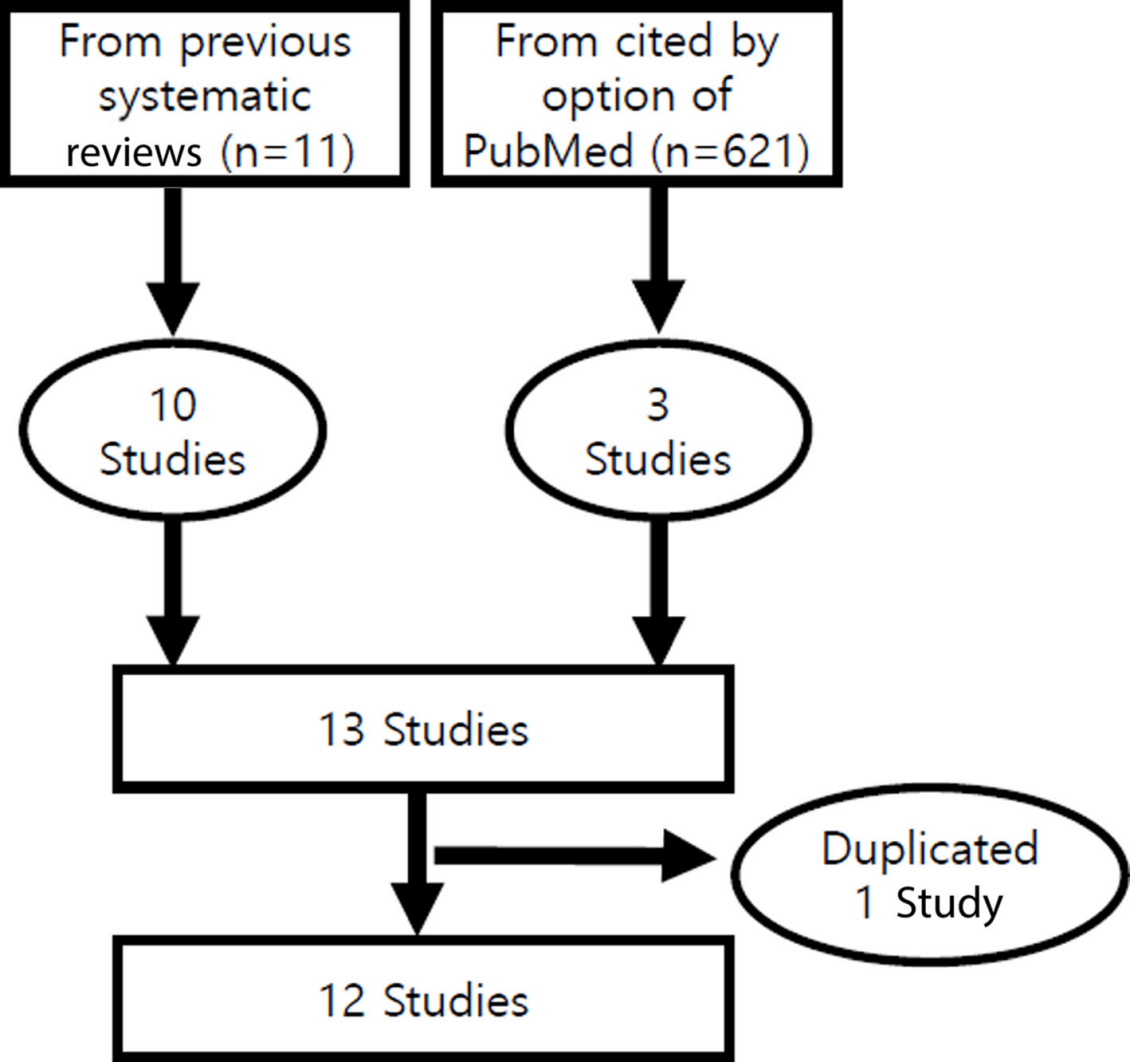
Flow chart of the final selection of follow-up studies

Among 11 articles selected by 4 systematic reviews in Table 1, Griffine et al. [16] was excluded because the follow-up outcome was not hypertension but systolic blood pressure. And a total of 621 studies cited the 11 studies, and 3 studies were also secured [28–30]. And Qi et al. [9] and Peng et al. [28] had the same participants, so Peng et al. [28] was excluded because it had fewer hypertension patients. Finally, 12 studies having 13 cohorts were selected [9, 12–21, 29, 30] (Fig. 1). When organized by sex, it is classified into 3 men, 3 women, and 7 sex-adjusted cohorts.

### Summary effect size

**Fig. 2:**
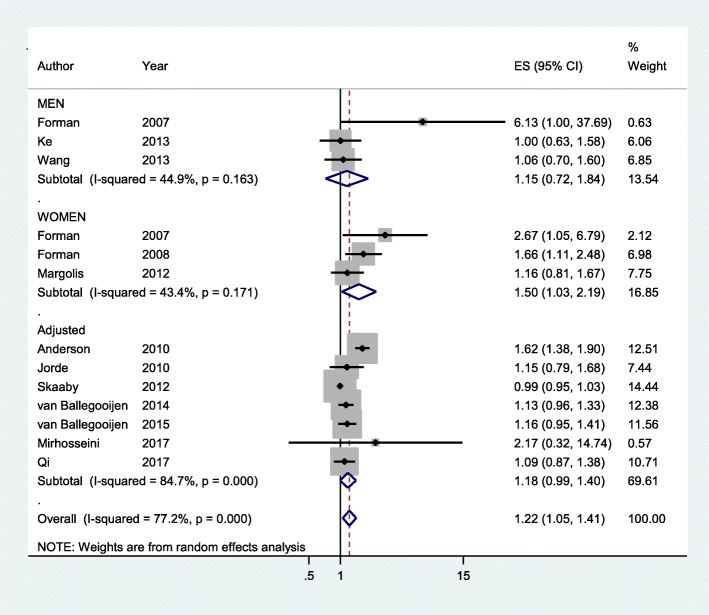
Forest plot (CI: confidence interval; ES: effect size)

**Fig. 3:**
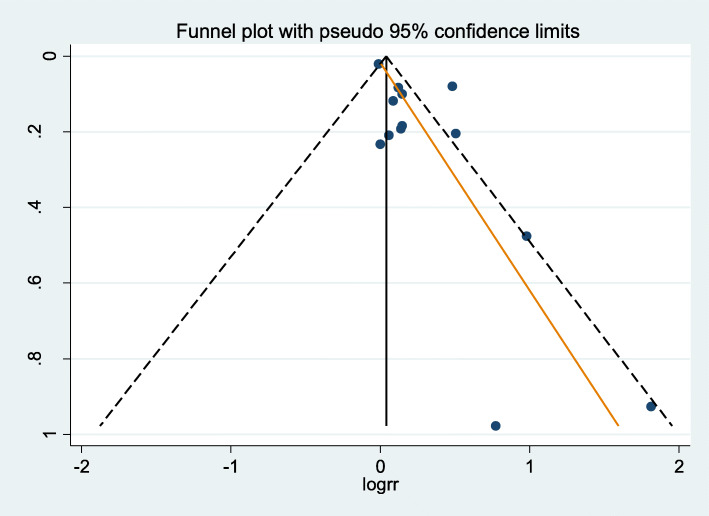
Funnel plot (*P*-value of Egger’s test =0.015; logrr: logarithm of relative risk; s.e.: standard error)

The meta-analysis of 13 cohorts resulted in the lower the vitamin D, the higher the risk of hypertension statistically significant (sRR, 1.22; 95% confidence interval [CI], 1.05 to 1.41; I-squared, 77.2%) (Fig. 2). The *P*-value of Egger’s test (=0.015) and asymmetry of funnel plot (Fig. 3) showed that there was a publication bias. And TFA resulted in that statistical significance disappeared in the association between vitamin D level and hypertension risk (filled sRR, 1.03; 95% CI. 0.89 to 1.18) (Table 2).

**Table 2:** Subgroup analysis by potential confounders^a^

	Random effects meta-analysis	Random method trim & filled analysis
All (*n* = 13)	1.22 (1.05–1.41) [77.2]	1.03 (0.89–1.18)
Adjusted (*n* = 7)	1.18 (0.99–1.40) [84.7]	1.02 (0.87–1.21)
Men (*n* = 3)	1.15 (0.72–1.84) [44.9]	1.15 (0.72–1.84)
Women (*n* = 3)	1.50 (1.03–2.19) [43.4]	1.16 (0.77–1.75)

^a^Summary relative risk (95% confidence interval) [I-squared value (%)]

### Subgroup analysis

In the subgroup analysis by 3 sex group, only women cohorts had statistical significance (sRR, 1.50; 95%CI. 1.03 to 2.19; I-squared, 43.4%) (Fig. 2). However, TFA in women cohorts resulted in that statistical significance disappeared (filled sRR, 1.16; 95% CI, 0.77 to 1.75) (Table 2).

## Discussion

### Comparison of results of previous systematic reviews

The main result was that a lower level of circulating 25(OH)D was associated with a significant, 1.22-fold (95% CI, 1.05 to 1.41) increase in hypertension risk in adults while publication bias exists. The above results obtained from 12 studies were like the results of Qi et al. [9] estimated from 7 studies (sRR = 1.24, 95%CI: 1.08–1.41). The I-squared value representing the level of heterogeneity was also similar (77.2% vs 72.1%).

However, the TFA considering publication bias resulted in that there was no association between vitamin D level and hypertension risk regardless of sex groups (Table 2). These results were like Zhang et al. [31]. They performed dose-response meta-analyses by sex. While the *P*-value of Egger’s test was 0.38, the hypertension risk per 25 nmol/L increments in 25(OH)D levels did not have statistical significance in men (95% CI, 0.85 to 1.00) and in women (95% CI, 0.76 to 1.01).

### Adjustment of publication bias

The funnel plot to check a publication bias showed asymmetry in this study. Only Kunutsor et al. [8] in Table 1 mentioned the funnel plot in Method. While they selected 6 studies for meta-analysis, they did not draw the plot based on that it was not useful in meta-analysis selecting less than five studies. Instead, they judged no evidence of publication bias by *P*-value of Egger’s test (=0.08). Thus, there is necessary to adjust the positivity criterion of presenting a publication bias in Egger’s test as *P*-value < 0.1 [32]. In addition, the *P*-value of Egger’s test in Burgaz et al. [7] containing 3 studies was 0.36. These facts illustrated that the *P*-value of Egger’s test decreased as the increased number of selected studies for meta-analysis.

In addition to publication bias, causes of funnel plot asymmetry involve chance, choice of effect measure, choice of precision measurement, and heterogeneity [33]. The selected cohorts were 12 studies, so that chance could be ruled out. And effect measures and precision measures could be rule out because the selected studies have the same study design.

### Strengths and Limitations

The major strength of this study was secured two additional studies [29, 30] so that the number of selected studies for meta-analysis could be over 10. This is one of four criteria for appropriateness of statistical tests for publication bias suggested by Ioannidis and Trikalinos [34]. Accordingly, it was possible to confirm publication bias by Egger’s test, and to provide bias-adjusted results by TFA [35]. In contrast, previous systematic reviews in Table 1 could not sufficiently consider publication bias and did not perform TFA.

The main limitation was related to the limitations of TFA. The effect size adjusted for publication bias provided by TFA should meet with the assumption that asymmetry in the funnel plot was only caused by publication bias [36]. Because this study could not rule out the asymmetry of funnel plot was made by heterogeneity [27]. But the bias-adjusted results by TFA having no statistically significant association between vitamin D levels and the risk of hypertension might be valid based on that publication bias is commonly associated with the inflated intervention effect [35]. In addition, TFA should be performed with great caution when using software programs [27]. Because there were 3 types of the estimator determining the number of points to trim in each iteration [27]. Author selected the linear estimator because it is stable in most situations and is the default in STATA software [26]. Another limitation was that different studies have different criteria for categorizing serum 25(OH)D levels. Therefore, the author performed a meta-analysis by extracting the outcomes of the lowest to the highest level.

## Conclusion

In conclusion, the bias-adjusted results by TFA had no statistically significant association between vitamin D levels and the risk of hypertension. The significant results in previous systematic reviews could be interpreted as due to publication bias.

## Data Availability

No data.

## Abbreviations

25(OD)D: 25-hydroxyvitamin D
CI: Confidence intervals
sRR: Summary relative risk
TFA: Trim-and-fill analysis
